# Complete Genome Sequence of a Rabies Virus Isolate from Cattle in Guangxi, Southern China

**DOI:** 10.1128/genomeA.00137-12

**Published:** 2013-01-31

**Authors:** Hai-Bo Tang, Xiao-Xia He, Yi-Zhi Zhong, Su-Huan Liao, Tao-Zhen Zhong, Lin-Juan Xie, Yan Pan, Zhuan-Ling Lu, Xian-Kai Wei, Yang Luo, Ting Rong Luo

**Affiliations:** State Key Laboratory for Conservation and Utilization of Subtropical Agro-Bioresources and College of Animal Sciences and Veterinary Medicine, Guangxi University, Nanning, China

## Abstract

A street rabies virus (RV) isolate, GXHXN, was obtained from brain tissue of rabid cattle in the Guangxi Zhuang Autonomous Region of China in 2009. GXHXN is the first isolate from cattle in China with its entire genome sequenced and is closely related to BJ2011E from horse in Beijing, WH11 from donkey in the Hubei Province, and isolates from dogs in the Guangxi and Fujian Provinces, with homologies of 97.6% to 99.6%. It is more distantly related to isolates from domestic cat, pig, Chinese ferret badger, and vaccine strains, with homologies of 83.1% to 88.0%.

## GENOME ANNOUNCEMENT

Rabies is a cosmopolitan zoonotic disease, which causes a fatal infection of the central nervous system. It results in acute, progressive, irreversible lethal encephalomyelitis and extremely serious psychosocial panic; globally it is responsible for more than 55,000 human deaths annually (1). In recent years, the reported human rabies cases reached a peak in 2007 (3,300 cases) in China (2), where the Guangxi Zhuang Autonomous Region is one of the traditional regions with high numbers of rabies outbreaks (2–4). A total of 4,031 human rabies cases were reported in Guangxi between 2000 and 2010, with peaks of more than 500 cases in 2003, 2004, and 2006.

Rabies virus (RV) belongs to the genus *Lyssavirus* in the family *Rhabdoviridae*. Its genome is a single-strand and negative-sense RNA of approximately 12 kb, encoding five structural proteins: nucleoprotein (N), phosphoprotein (P), matrix protein (M), glycoprotein (G), and RNA-dependent RNA polymerase (L) (5).

The street RV GXHXN was isolated from rabid cattle in Guangxi in 2009 by intracerebral injection into adult Kunming mice. Then, total RNA was extracted from infected mouse brain using TRIzol LS reagent (Invitrogen, Carlsbad, CA). Thirteen pairs of primers were designed based on the genomic sequence of the ERA strain (6) and used to amplify the whole genome of the isolate GXHXN. The PCR products were purified using an aqua-Spin gel extraction mini kit (Watson Biotechnologies, Inc.) and cloned into a pMD18-T vector (TaKaRa). Cloned DNA fragments were sequenced by the Sangon Biotech Co., Ltd. (Shanghai), and the genomic sequence was assembled by using SeqMan software (DNAStar Inc.). The complete genome of GXHXN has 11,923 nucleotides (nt), with a GC content of 44.75%. The coding sequences are as follows: 1,353 nt for the N gene, 894 nt for the P gene, 609 nt for the M gene, 1,575 nt for the G gene, and 6,387 nt for the L gene; the L gene has a double start codon (ATGATG). Sequence comparison indicates that the isolate GXHXN belongs to “group II” (7) and is closely related to BJ2011E isolated from horse in Beijing, WH11 isolated from donkey in the Hubei Province, and isolates GX219, GXLA11, GX074, and FJ008 from dogs in the Guangxi and Fujian Provinces, with homologies of 97.6% to 99.6% (7, 8). However, the isolate GXHXN also shares homologies of 83.1% to 88.0% with isolates from domestic cat, pig, Chinese ferret badger, and vaccine strains (6, 9–12).

The viral G protein is a major contributor to RV pathogenicity (13, 14). The G protein of isolate GXHXN is the same as in other virulent strains, with amino acids Ala242, Asp255, Ile268, and Arg333 involved in viral pathogenicity (15, 16). Although GXHXN was isolated from cattle, the complete genome analysis revealed that it is possibly derived from dog, and this finding will help in the exploration of the molecular mechanism of interspecies transmission of rabies.

### Nucleotide sequence accession number.

The complete genome sequence of GXHXN has been deposited in GenBank under accession number KC169986.
